# Disclosing a diagnosis of autism spectrum disorder without intellectual disability to pediatric patients in Japan in early diagnostic stages and associated factors: a cross-sectional study

**DOI:** 10.1186/s13030-022-00247-0

**Published:** 2022-08-20

**Authors:** Hiroyuki Sato, Misao Fujita, Atsushi Tsuchiya, Taichi Hatta, Katsumi Mori, Eisuke Nakazawa, Yoshiyuki Takimoto, Akira Akabayashi

**Affiliations:** 1 Department of Biomedical Ethics, Faculty of Medicine, University of Tokyo, 7-3-1 Hongo, Bunkyo-ku, Tokyo, 113-0033 Japan; 2 Uehiro Research Division for iPS Cell Ethics, Center for iPS Cell Research and Application, Kyoto University, 53 Seigoin Kawahara-cho, Sakyo-ku, Kyoto, 606-8507 Japan; 3 Institute for the Advanced Study of Human Biology (WPI-ASHBi), KUIAS, Kyoto University, Yoshidakonoe-cho, Sakyoku, Kyoto, 606-8501 Japan; 4 Kansai University Graduate School of Sociology, 3-3-35 Yamate-cho, Suita-shi, Osaka, 564-8680 Japan; 5 Graduate School of Public Health, Shizuoka Graduate University of Public Health, 4-27-2 Kita Ando, Aoi-ku, Shizuoka, 420-0881 Japan; 6 Division of Medical Ethics, New York University School of Medicine, 227 East 30th Street, New York, NY 10016 USA

**Keywords:** Autism spectrum disorder, Diagnosis disclosure, Quantitative research, Multivariate logistic regression analysis, Japan, Cross-sectional study

## Abstract

**Background:**

With a recent increase in the prevalence of autism spectrum disorder (ASD), an important issue has emerged in clinical practice regarding when and how patients themselves should be given explanations following a diagnosis of ASD. The clinical guidelines of the UK National Institute for Health and Care Excellence state that children diagnosed with ASD should receive an explanation about what ASD is and how it affects their development and functioning—“if appropriate”. However, the guidelines do not provide any specifics regarding what constitutes “appropriate” situations

**Methods:**

We conducted an anonymous self-administered postal questionnaire survey targeting all members of the Japanese Society for Child and Adolescent Psychiatry (*n*=1,995). The analysis included only physicians who had newly diagnosed pediatric patients with ASD in the past year. We imposed a limit of one year because diagnoses further back than that are difficult to recall; in other words, this would enhance the recall bias

**Results:**

The recovery rate was 30.8%, and the rate of diagnosis disclosure to patients themselves without intellectual disability was 15.3%. We asked 361 physicians who responded that “deciding on a case-by-case basis” was the ideal way to disclose an ASD diagnosis about 20 items prioritized by physicians at the time of diagnosis disclosure and extracted three factors through exploratory factor analysis. Multiple logistic regression analysis was performed with physician attributes, awareness of ASD as a disorder or personality, and the three extracted factors as explanatory variables; diagnosis disclosure was the dependent variable. The patient age group and only one of the three factors (i.e., “factor related to readiness to accept diagnosis”) showed a significant association with disclosure of the diagnosis to the individual. Items included in the “factor related to readiness to accept diagnosis” were as follows: the degree of parental understanding, relationship of the patient with their parents/physician, agreement in opinion between parents, parental consent, “sufficient” patient understanding, symptom stabilization, and a guarantee of sufficient time required to explain the diagnosis to the patient

**Conclusion:**

In clinical settings, disclosing an ASD diagnosis with the consideration of patient/parent readiness toward accepting the diagnosis could help to guide physicians in determining an ideal timing for disclosure. Future studies are needed to establish detailed and concrete guidelines regarding disclosure of an ASD diagnosis to patients.

**Supplementary Information:**

The online version contains supplementary material available at 10.1186/s13030-022-00247-0.

## Introduction

With a recent increase in the prevalence of autism spectrum disorder (ASD) [1], determining the timing and method of providing explanations to patients following their ASD diagnosis has become an issue of importance in clinical practice. According to clinical guidelines issued by the UK National Institute for Health and Care Excellence regarding ASD in patients aged < 19 years [2], when children are diagnosed with ASD, the physician should inform their parents/guardians to discuss diagnostic results and, “if appropriate,” explain to the patients themselves what ASD is and how it affects their development and functioning. However, the guidelines do not specify what constitutes an “appropriate” situation.

The National Autistic Society in the UK has published “A guide for disclosing ASD to parents and carers of children with ASD,” which mentions that children with ASD have the right to know their diagnosis, and that although not telling the children about the diagnosis may seem like a thoughtful decision, it becomes more difficult to reveal the information as time passes [3].

The disclosure of an ASD diagnosis is important in order for children to develop their own identity, construct their social relationships, and control their lives skillfully [4]. Moreover, if they are diagnosed, they can receive relevant public services. However, while this could be advantageous, there is also a potential disadvantage of experiencing prejudice [4].

To date, few studies have examined disclosure rates for ASD diagnosis. Of the surveys conducted in English-speaking countries, a Canadian online survey targeting 133 parents and an Irish interview survey targeting 7 parents both reported that roughly 70% of children were informed of their diagnosis by their parents [5, 6]. In Japan, previous studies reported variable rates of diagnosis disclosure, ranging from 2% in a medical record survey at a university hospital [7] to 65% in a questionnaire survey at a clinic specialized in developmental disorders [8].

In clinical research, we primarily focus on the following question: At what timing and under what conditions do physicians think it is appropriate to tell their patients about their disease, and what are their actual disclosure behaviors? In the present study, we aimed to clarify specifically what physicians think are relevant points to consider when disclosing an ASD diagnosis to their patients. In other words, the main purpose of this study was to present a concrete definition of an “appropriate” situation. To this end, we conducted an exploratory fact-finding and awareness questionnaire survey among physicians who diagnose and treat ASD patients regarding the ideal ways of disclosing the diagnosis to patients, as well as items they prioritize at the time of disclosure. We then analyzed factors related to the disclosure of an ASD diagnosis to patients.

## Methods

### Questionnaire survey

An anonymous self-administered postal questionnaire survey was conducted in November 2015, targeting all members of the Japanese Society for Child and Adolescent Psychiatry (*n* = 1,995). Questionnaire items were created by the authors after referring mainly to studies previously conducted in Japan [9–14], given that the survey was to be conducted in Japan. These items were then modified according to expert opinions provided by six pediatric psychiatrists with ≥ 10 years of clinical experience (Additional file 1)**.**

In addition to items regarding physician attributes, the questionnaire asked about 1) Current reality: the number of newly diagnosed ASD patients in the past year (November 2014—October 2015) (We imposed a limit of one year because diagnoses further back than that are difficult to recall; in other words, this would enhance the recall bias), the patient age groups (up to elementary school / junior school and higher), and the number of patients/parents who were notified of the diagnosis; as well as 2) Awareness: ideal ways to disclose ASD diagnosis (disclose as a general rule/case-by-case/do not disclose as a general rule), and whether they consider ASD a disorder or personality trait.

Next, we asked about items prioritized by physicians when disclosing the diagnosis (20 items; assessed on a 4-point Likert scale). These items were selected by referring to previous studies [9–14], while also taking into consideration situations in which physicians are advised to refrain from telling children with developmental disorders about these [15], and factors that are prioritized when explaining Duchenne’s muscular dystrophy to children with this disease [16] (similarly, these items were revised according to expert opinions).

ASD was defined according to the Diagnostic and Statistical Manual of Mental Disorders Fifth Edition (DSM-5). In addition, diagnoses that existed pre-DSM-5 (e.g., pervasive developmental disorder, autism, Asperger’s syndrome) were also included in the definition of ASD. “Patient” was defined as a “high functioning patient without intellectual disability (with an IQ of about 70 or higher),” while “pediatric” was defined as “age < 18.”

### Analysis methods

First, a simple tabulation was performed for physician attributes, the status of ASD diagnosis and disclosure in the past year, and physician awareness regarding ASD and its disclosure.

The rate of disclosure (for patients and parents) was calculated from the number of pediatric patients who were newly diagnosed with ASD by their physicians in the past year, and the number of patients/parents who were informed about the ASD diagnosis. If the number of patients diagnosed by a single physician exceeded the mean number + 3SD, these cases were excluded from subsequent analyses.

To confirm that the respondents in the present study were similar to and therefore representative of the overall physician population, we conducted a chi square goodness-of-fit test of distribution for all society members and for respondents with regard to age (5 − year increments), sex (male/female), specialty (pediatrician, psychiatrist, etc.), and region.

Next, with respect to the ideal ways of disclosing the diagnosis, we extracted the group of respondents that selected “case-by-case” (“case-by-case group”), based on the assumption that these respondents are likely to change their stance on whether or not to notify their patients for various reasons (case-dependent) in an attempt to improve the disclosure process. We reasoned that focusing on the case-by-case group would enable us to analyze more clearly the factors prioritized by physicians when disclosing an ASD diagnosis.

With respect to the 20 items (i.e., items prioritized by physicians when disclosing an ASD diagnosis), the responses (“I don’t consider it important,” “I don’t consider it very important,” “I consider it somewhat important,” and “I consider it important”) were rated on a scale of 1 to 4, and Polychoric correlation coefficients [17] were determined from these scores. Data were then analyzed using the maximum-likelihood method and exploratory factor analysis with promax rotation [18].

Finally, multiple logistic regression analysis was performed with physician attributes, specialty, patient age group, whether ASD is considered a disorder or personality, and three factors prioritized by physicians in disclosing an ASD diagnosis (from factor analysis) as explanatory variables, and the presence/absence of diagnosis disclosure as the objective variable.

All analyses were performed using SAS version 9.4 (SAS Institute Inc., Cary, NC, USA), with *p* < 0.05 considered statistically significant. This study was approved by Research Ethics Committee of the Faculty of Medicine of the University of Tokyo (review No. 10950).

## Results

### Recovery rate

Responses were obtained from 612 of 1,995 physicians to whom questionnaire forms had been sent; 9 forms were returned due to an unknown address (recovery rate: 30.8%). Of the 612 physicians, 463 who responded that they had newly diagnosed pediatric (age < 18 years) patients with ASD in the past year were included in the analysis.

### Simple tabulation

The mean age of physicians who responded to the questionnaire was 48.7 years, and 54.1% were male. Roughly half of the physicians worked in clinical psychiatry treating primarily pediatric patients, with a mean number of years in ASD practice of 14.4 years. The mean number of pediatric patients who were newly diagnosed with ASD in the past year was 41.2, and 60.8% of physicians informed the patients themselves that they had ASD. With regard to disclosing diagnoses to pediatric patients, 79.2% of physicians considered it appropriate to inform them on a case-by-case basis, and 53.0% regarded ASD as a personality trait (Table 1).

**Table 1:** Characteristics of respondents (*n* = 463)

		Number of physicians (%) or mean value (range)
Age (years)		48.7 (28 – 83)
Sex	Male	250 (54.1)
	Female	212 (45.9)
Specialty	Pediatrics	110 (24.1)
	Psychiatry (pediatric)	236 (51.8)
	Psychiatry (adult)	107 (23.5)
	Other	3 (0.7)
Specialty (multiple responses)	Pediatrician	132 (28.5)
	Psychiatrist	261 (56.4)
	Designated physician of mental health	276 (59.6)
	Japanese Society for Child and Adolescent Psychiatry-certified physician	87 (18.8)
	None	43 (9.3)
Workplace	University hospital	68 (14.7)
	General hospital	62 (13.4)
	Psychiatric hospital	97 (21.0)
	Children’s hospital	30 (6.5)
	Clinic	125 (27.1)
	Child consultation center	9 (1.9)
	Public health center/Mental health and welfare center	6 (1.3)
	Child developmental center	46 (10.0)
	Other	19 (4.1)
Years of experience as a physician		22.2 (4–58)
Length of ASD practice (years)		14.4 (1 – 51)
Number of pediatric patients diagnosed with ASD in the past year		41.2 (1 – 900)
Patients in elementary school and lower	Diagnosed with ASD	408 (88.1)
	Not diagnosed with ASD	55 (11.9)
Patients in junior high school and higher	Diagnosed with ASD	409 (88.3)
	Not diagnosed with ASD	54 (11.7)
Disclosure to patients	Disclosed	253 (60.8)
	Not disclosed	163 (39.2)
Ideal diagnosis disclosure	Disclose as a general rule	78 (17.1)
	Do not disclose as a general rule	17 (3.7)
	Decide on a case-by-case basis	361 (79.2)
Is ASD a disorder or personality	100% personality	10 (2.3)
	more like personality	223 (50.7)
	more like disorder	186 (42.3)
	100% disorder	21 (4.8)

Age (years), length of ASD practice (years), and number of pediatric patients diagnosed with ASD are presented as a mean (range); other items are shown as number (%). The total number may not always match the number of study participants, as there were missing responses for some items

A chi-square goodness-of-fit test for all 1,995 members of the Japanese Society for Child and Adolescent Psychiatry (the overall population) and the 612 members who responded to the survey revealed no significant differences (sex: *p* = 0.437, specialty: *p* = 0.524, region: *p* = 0.997, age: *p* = 0.998), signifying that the distribution of the respondents did not deviate from the distribution of the overall population (Additional file 2).

### ASD diagnosis disclosure rate

Among the 463 physicians who responded that they had diagnosed pediatric patients with ASD, 442 specified the number of diagnosed patients. Of these, four physicians with the highest numbers of diagnosed patients (more than the mean + 3 SD, i.e., 250 patients) were considered ‘extreme’ respondents who diagnose ≥ 20 first-visit patients every month; these were excluded from subsequent analyses. Consequently, the total number of pediatric patients diagnosed with ASD annually was 15,884, with a mean number per physician (*n* = 438) of 36.3 (43.1 SD). ASD diagnosis disclosure rates to pediatric patients and their parents were 15.3% and 85.3%, respectively.

### Extraction of “case-by-case” respondents and exploratory factor analysis

**Fig. 1:**
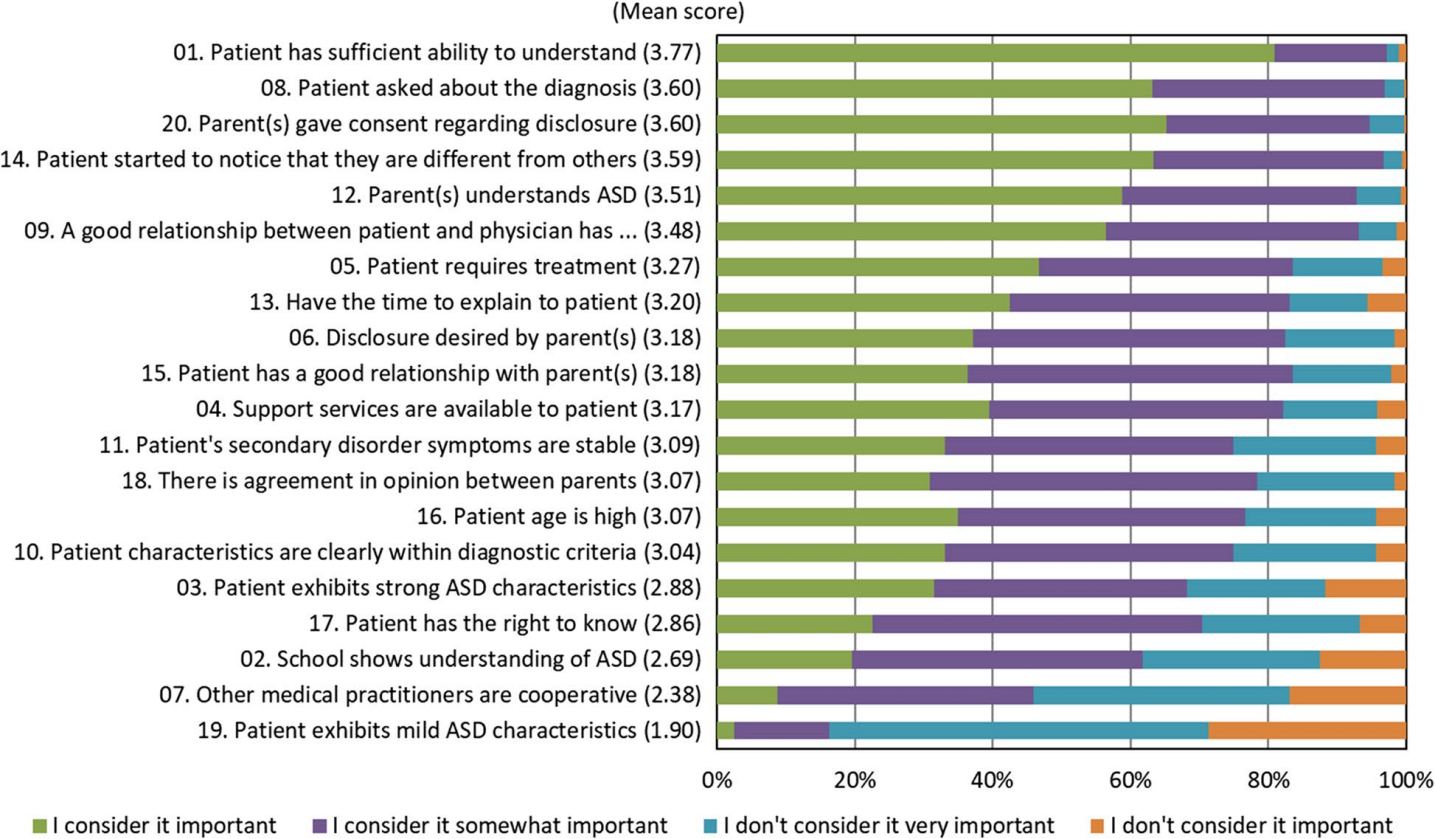
Items prioritized by physicians when disclosing an ASD diagnosis

We extracted the 361 physicians who chose “case-by-case” as their ideal method of disclosure, and made a simple tabulation of their responses regarding 20 items on which physicians place importance when disclosing an ASD diagnosis (Fig. 1). Figure 1 shows the mean score for each item when physician responses are rated on a scale of 1 (“I don’t consider it important”) to 4 (“I consider it important”). The six items prioritized most by physicians when disclosing ASD diagnosis, in descending order, were “the patient has sufficient ability to understand,” “the patient asked [about the diagnosis],” “parent(s) gave consent regarding disclosure,” “the patient started to notice they are different from others,” “parent(s) showed understanding,” and “a good relationship between the patient and physician has been established” (mean score: ≥ 3.5 points). “Older age" was ranked 14^th^.

We then performed exploratory factor analysis with promax rotation and the maximum likelihood method using the 20 items. Three factor solutions were adopted from the scree plot, and the factor score was calculated for each. Taking into consideration the items with high factor loading, the three factors were named as follows: “factor related to readiness to accept diagnosis,” “factor related to treatment systems,” and “factor related to needs for disclosure” (Table 2).

**Table 2:** Results of exploratory factor analysis

	Factor		
	I	II	III
12. Parent(s) understands ASD	0.848	-0.055	0.043
15. Patient has a good relationship with parent(s)	0.766	0.043	0.035
11. Patient's secondary disorder symptoms are stable	0.688	0.001	-0.185
09. A good relationship between patient and physician has been established	0.522	0.155	0.089
18. There is agreement in opinion between parents	0.468	0.107	0.207
13. Have the time to explain to patient	0.461	0.001	0.283
20. Parent(s) gave consent regarding disclosure	0.460	-0.054	0.364
01. Patient has sufficient ability to understand	0.227	0.168	0.076
07. Other medical practitioners are cooperative	0.093	0.621	-0.061
04. Support services are available to patient	0.056	0.504	0.103
05. Patient requires treatment	-0.137	0.494	0.370
02. School shows understanding of ASD	0.357	0.476	-0.312
03. Patient exhibits strong ASD characteristics	-0.037	0.470	0.094
19. Patient exhibits mild ASD characteristics	0.066	0.449	-0.017
10. Patient characteristics are clearly within diagnostic criteria	0.001	0.395	0.304
08. Patient asked about the diagnosis	0.094	-0.114	0.538
14. Patient started to notice they are different from others	0.351	-0.133	0.506
17. Patient has the right to know	-0.113	0.258	0.435
06. Disclosure desired by parent(s)	0.013	0.197	0.409
16. Patient age is high	0.066	0.310	0.335
Inter-factor correlation	I	II	III
I: Readiness to accept diagnosis	—	0.326	0.248
II: Treatment systems		—	0.154
III: Needs for disclosure			—

Among 361 physicians who responded that "deciding on a case-by-case basis” was the ideal way of disclosing an ASD diagnosis, 324 who provided responses to all 20 items were included in this analysis. Four physicians who diagnosed ≥ 250 patients with ASD in the past year were excluded

### Multiple logistic regression analysis

Multiple logistic regression analysis, which was performed with the presence/absence of diagnosis disclosure as the objective variable, revealed significant associations for patient age group and the “factor related to readiness to accept diagnosis” (Table 3). Physicians who saw patients in the junior high school and older age group disclosed an ASD diagnosis more readily (Adjusted odds ratio, AOR: 12.762), and the doctor who gives more consideration for “factor related to readiness to accept diagnosis” were more likely not to disclose an ASD diagnosis to the individual (AOR: 0.606). In addition, while a statistically significant difference was not found, the odds ratio for pediatric psychiatrists over pediatricians was higher (AOR: 1.918, *p* = 0.053), demonstrating a higher tendency for pediatric psychiatrists to disclose an ASD diagnosis to the patients.

**Table 3:** Results of multiple logistic regression analysis

	AOR	95% CI	*p* value
Male – ref. Female	0.713	0.410 – 1.239	0.230
Years of experience in ASD practice	0.973	0.946 – 1.001	0.061
Psychiatry (pediatric) – ref. Pediatrics	1.918	0.992 – 3.708	0.053
Psychiatry (adult) – ref. Pediatrics	0.921	0.389 – 2.176	0.850
Examine patients in elementary school and lower	0.812	0.301 – 2.187	0.680
Examine patients in junior high school and higher	12.762	4.030 – 40.415	< 0.001
ASD is a personality trait	1.113	0.658 – 1.885	0.689
I. Factor related to readiness to accept diagnosis	0.606	0.419 – 0.878	0.008
II. Factor related to treatment systems	0.972	0.689 – 1.371	0.872
III. Factor related to needs for disclosure	1.049	0.739 – 1.489	0.789

*AOR* adjusted odds ratio, *CI* confidence interval

Among 361 physicians who responded that "deciding on a case-by-case basis” was the ideal way of disclosing ASD diagnosis, 288 who provided responses to all items were included in this analysis. Four physicians who diagnosed ≥ 250 patients with ASD in the past year were excluded. Hosmer–Lemeshow's goodness of fit test: *p* = 0.4635

## Discussion

### Factors related to disclosure of ASD diagnosis

#### Patient age

Previous studies [11, 19] reported that diagnosis disclosure rates increase with patient age. In the present study as well, patient age was associated with the disclosure of an ASD diagnosis. However, whereas “the level of understanding” was ranked highest in terms of mean score among the 20 items prioritized by physicians when disclosing an ASD diagnosis, “age” was ranked 14th. Based on these results, it is predicted that physicians make judgments as to whether to reveal a diagnosis to patients or not while observing their level of understanding—which is expected to increase with age—rather than considering their actual age as a factor.

The level of understanding, an indicator of growth, differs from patient to patient. Age, on the other hand, is an objective indicator that is unlikely to serve as an absolute condition when determining when/how to inform patients of a diagnosis. Physicians should thus consider and evaluate each patient’s degree of maturity when disclosing the diagnosis.

#### Specialty

While a significant difference was not demonstrated, relative to pediatricians, pediatric psychiatrists appeared to disclose an ASD diagnosis more frequently. One possible reason for this is that more patients visit psychiatric departments with secondary disorders such as depression and anxiety and require medication or other treatments. In other words, patients might receive an explanation about ASD as the cause of the disorder(s) when they begin treatment for a secondary disorder.

Second, the physician’s hours of practice in pediatrics vs. psychiatry may have affected the results. The proportions of respondents who selected “I consider it important” and “I consider it somewhat important” for the item, “Have the time to explain to the patient him/herself,” were 69.3% in pediatrics, and 52.6% and 46.6% in psychiatry (pediatric and adult, respectively), according to the stratified results. However, no data were available to enable a direct comparison of the consultation hours. We speculate that pediatricians might be more hesitant to disclose diagnoses to patients, thinking they lack sufficient time to explain.

#### Whether ASD is a disorder or personality

Nearly half of the physicians engaged in clinical practice considered ASD a personality trait rather than a disorder, as revealed by the question regarding physician awareness. Multiple logistic regression analysis revealed no significant association between views on ASD and diagnosis disclosure, and physicians appeared to tell their patients that they have ASD, either as a personality trait or disorder, based on their own reasoning (or for other reasons). These findings suggest the need to discuss ideal ways of disclosing an ASD diagnosis to pediatric patients in the future.

#### Factor related to readiness to accept diagnosis

The most intriguing and novel finding of the present study is that physicians who prioritized the “factor related to readiness to accept diagnosis” at the time of diagnosis disclosure were more likely to withhold disclosure of the diagnosis (AOR = 0.606).

Items included in the “factor related to readiness to accept diagnosis” are summarized in Table 2; namely, the degree of parent understanding, patient to parent/physician relationship, agreement in opinion between parents, parental consent, “sufficient” patient understanding, symptom stabilization, and a guarantee that they have the time to explain.

At first glance, it might seem intuitive to imagine that physicians who prioritize the “factor related to readiness to accept diagnosis” are more likely to tell their patients that they have ASD. However, since the present study only examined initial diagnosis cases (i.e., newly diagnosed ASD patients) within the 1-year period, it is expected that physicians had not grasped sufficiently the degree of parental understanding, the relationship between the patient and their parents/physician, agreement in opinion between parents, and “sufficient” patient understanding. More than anything, 1 year is not likely enough time for physicians to establish a solid relationship of trust with the patient/family. We suspect that it is for these reasons that physicians are more cautious about informing patients of their diagnosis in the early stages following diagnosis.

Our results suggest that many physicians consider telling a patient about the diagnosis of ASD once they have established a relationship of trust with the patient and their family, with consideration of the patient’s growth (sufficient understanding) as well as the degree of parental understanding. While the presence/absence of support services and the need for treatment (i.e., environmental factors) are obviously of clinical importance, the implication of our findings is that those factors were not directly associated with “disclosing an ASD diagnosis to patients.” In other words, reasons such as “services are available” or “treatment is necessary” alone are not strong factors that should necessarily lead to the disclosure of an ASD diagnosis to patients.

In summary, the “factor related to readiness to accept diagnosis” was strongly associated with the disclosure of an ASD diagnosis by physicians. In order to further verify this finding, the next step is to conduct a cross-sectional study including physicians who have been engaged in ASD practice for longer than one year. In addition, a longitudinal study should be conducted, in which physicians are followed from the time of a new ASD diagnosis up to diagnosis disclosure, asking them to provide in detail the reasons for disclosure. Furthermore, a qualitative study including, for example, semi-structured interviews with physicians who disclosed an ASD diagnosis to their patients, will be necessary.

Lastly, we arrived at the following hypothesis from the present exploratory study: “For physicians, the appropriate timing for disclosing ASD diagnosis to a patient is when the patient’s parents have a higher degree of understanding and the physician has established a good relationship with the patient and their parents.” We would like to conduct other studies in the future to verify this hypothesis.

### Limitations

This study has several limitations worth noting. First, the recovery rate was low (30.8%). However, results for the chi-square goodness-of-fit test showed that the distributions of the attributes of the respondents did not vary from those of the society as a whole (the overall population), suggesting that the subjects in the present study are at least somewhat representative of the overall population of physicians.

Second, as the diagnosis disclosure rate was calculated from the number of pediatric patients with ASD and that of patients who were informed of their diagnosis by the respondents in the past year, recall bias is possible. Third, since the disclosure rate was based on the number of patients diagnosed by physicians, different physicians might have diagnosed the same patient. Fourth, because cases examined in this study were limited to those in which patients were given their diagnosis in the initial stages, we were unable to ascertain disclosure situations among patients who saw a doctor for longer than one year. Finally, as the present questionnaire survey was conducted in 2015, and a substantial amount of time has passed since then, it may not reflect the most current reality. Publication of the survey was delayed due to some necessary troubleshooting of the analysis method to improve suitability, and even more by the COVID-19 pandemic, which created inconveniences in the research environment. However, as stated in the main text, a longitudinal follow-up study has been planned for 10 years post-survey, and the present study findings can be used as preliminary reference data therein.

## Conclusions

We clarified the actual conditions under which a diagnosis of ASD is disclosed to a patient in the initial stage of diagnosis in Japan, as well as associated factors, among physicians who disclose the diagnosis to patients on a case-by-case basis. Although relationships between some items and diagnosis disclosure to patients have been suggested by previous studies, the present study findings are remarkable in that analyses were performed by adjusting for most of those explanatory variables. In clinical settings, our findings may offer a sort of guidance for physicians to determine the timing of disclosure while considering the factors related to readiness to accept diagnosis for the patients newly diagnosed in the past year. Further cross-sectional, longitudinal, and qualitative studies are anticipated towards the establishment of detailed and concrete guidelines regarding the disclosure of an ASD diagnosis to patients.

## Supplementary Information

**Additional file 1.** 

**Additional file 2.**  

## Data Availability

The datasets analyzed in the current study are available from the corresponding author upon reasonable request.

## Abbreviation

ASD: Autism spectrum disorder
