# Supplementary material for: Disclosing a diagnosis of autism spectrum disorder without intellectual disability to pediatric patients in Japan in early diagnostic stages and associated factors: a cross-sectional study
Source: Biopsychosoc Med. 2022 Aug 20;16:18. doi: 10.1186/s13030-022-00247-0 (PMC9391641; doi:10.1186/s13030-022-00247-0)
Supplement: Supplementary file 1 — Additional file 1. [file 13030_2022_247_MOESM1_ESM.docx]

**Survey on Notification of Autism Spectrum Disorder (ASD):**

**Notification to Pediatric Patients Themselves**

<Definition of terms in the survey>

• **Autism Spectrum Disorder (hereinafter referred to as ASD)** is defined in the DSM-5 (Diagnostic and Statistical Manual of Mental Disorders Fifth Edition), but on this questionnaire, unless specially noted, it is used to include things continuing from before the DSM-5 as well (e.g., pervasive developmental disorder, autism, Asperger’s syndrome).

• **Patient** refers to **high-functioning patients without mental retardation (IQ of roughly 70 or above)**.

• **Child/ren** refers to **up to high school students (under age 18)**.

• **Characteristics** does not include the diagnosis itself, but refers to symptoms of ASD the child has.

Please read question items carefully, and circle the appropriate number or fill in the blank ( ) area with a number or words

.

Please enclose the questionnaire in the self-addressed stamped envelope, and

mail **by November 30, 2015 (Mon)**.

Thank you.

Contact: 7-3-1 Hongo, Bunkyo-ku, Tokyo-to 113-0033

Department of Biomedical Ethics, Graduate School of Medicine, The University of Tokyo

Principal investigator: Yoshiyuki Takimoto

Corresponding author: Hiroyuki Sato

Tel: 03-5841-3618 (Mon-Wed-Fri 11:00-17:00)

(Tue-Thu 15:00-17:00)

FAX: 03-5841-3319

　　　　　　　　　　　　　　　　　　　E-mail: [hirosato@m.u-tokyo.ac.jp](mailto:hirosato@m.u-tokyo.ac.jp)

|  |  |  |  |
| --- | --- | --- | --- |

**[Question 1] We will ask you about yourself and your place of employment.**

**(Please circle the appropriate number or fill in the blank with a number or words)**

A. Gender (select one) 1. Male 2. Female

B. Age (fill in a number) ( ) years

C. Primary department (select one)

1. Pediatrics 2. Psychiatry (pediatric) 3. Psychiatry (adult) 4. Psychosomatic medicine

5. Junior residency 6. Basic research 7. Other ( )

D. Medical specialties, etc. held **(can select multiple)**

1 Japan Pediatric Society: Pediatric specialist　　　　　　2 Japanese Society of Psychiatry and Neurology: Psychiatry specialist

3 Designated physician of mental health　　　　　　　　　　　　　4 Japanese Society for Child and Adolescent Psychiatry: Certified physician

5 Do not hold any of the above medical specialties, etc.

E. Years of experience as a physician (fill in a number) ( ) years

F. Years of experience involved in ASD diagnosis and treatment (fill in a number) ( ) years

G. Place of employment (if there are two or more, the place of employment with the most diagnosis and treatment of ASD children **(select one)**

1. University hospital　　　　　　　　　2. General hospital　　　　　　　　　　　3. Psychiatric hospital

4. Children’s hospital　　　　　　　5. Clinic, medical office　　　　　　6. Child consultation center

7. Public health center/mental health and welfare center　　　　8. Habilitation center

9. Other ( )

H. Location of the place of employment that was the answer for Question G (fill in one prefecture name) ( )

I. In the area around the place of employment that was the answer for Question G, is the access to services (habilitation, special education classrooms, etc.) that can be received by patients themselves that have been diagnosed with ASD good or bad? (select one)

1. If I had to say, good　　　2. If I had to say, bad　　　3. Neither

　　　 　　I-2. This question is asked for **those who responded with answer 1 (If I had to say, good) to Question I**.

　　　　　　What specific services have good access? **(can select multiple)**

　　　　　　1. Habilitation　　　　　　　　2. Resource room classes/special education classroom　　　　3. Special education school

　　　　　　4. Education center　　　　5. Employment support organization　　　　6. Developmental disabilities support center

7. Organization for those affected

　　　　　　8. Other ( )

**[Question 2] We will ask you about ASD diagnoses over the past year (November 2014 to October 2015).**

A. In **the past year (November 2014 to October 2015)**, did you have any **patients** **newly** diagnosed with ASD?

(select one)

1. No **patients** diagnosed　　　　　　　　Please proceed to **page 4 question 4**

2. **Patients** diagnosed

　　　　　　　　　Please proceed to **the next question, Question B**

B. Please denote **all** diagnostic manuals used during the above period. **(can select multiple)**

1. DSM-5　　2. DSM-IV-TR　　3. ICD-10　　4. Other ( )

C. Please select **all** age groups of patients diagnosed with ASD during the above period. **(can select multiple)**

1. Preschool　　2. Elementary school student　　3. Middle school student　　4. High school student　　5. College student/adult

D. Please select **the most common** age group of patients diagnosed with ASD from previous Question C. **(select one)**

1. Preschool　　2. Elementary school student　　3. Middle school student　　4. High school student　　5. College student/adult

E. During the above period, how many **pediatric patients (up to high school) were newly diagnosed with ASD?**

Please recall and record the number as accurately as possible. ( ) patients **(fill in a number)**

If there were **0** **pediatric patients** diagnosed for previous Question E　　　　Please proceed to **page 4 question 4**

If there was **at least one** **pediatric patient** diagnosed for previous Question E

　　　　　　　　　Please proceed to **the next question, Question F**

F. **For how many of the pediatric patients from Question E** did you communicate the following items to **the parent(s)**?

(fill in the number of patients **for each item**)

F-1. I communicated **the diagnosis** to **the parent(s)** for ( ) of the patients from Question E.

F-2. I communicated **the patient’s ASD characteristics** to **the parent(s)** for ( ) of the patients from Question E.

F-3. I communicated **common ASD symptoms** to **the parent(s)** for ( ) of the patients from Question E.

G. **For how many of the pediatric patients from Question E** did you communicate the following items to **the pediatric patient themselves**?

(fill in the number of patients **for each item**)

G-1. I communicated **the diagnosis** to **the patient themselves** for ( ) of the patients from Question E.

G-2. I communicated **the patient’s ASD characteristics** to **the patient themselves** for ( ) of the patients from Question E.

G-3. I communicated **common ASD symptoms** to **the patient themselves** for ( ) of the patients from Question E.

H. During the above period, did you ever give a child who fell slightly short of the ASD diagnostic criteria a diagnosis with the goal of receiving services? (select one, if yes, fill in the number of patients)

About a third of the way done

**Please proceed to the next page**

1. No　　　　2. Yes ⇒ ( ) patients

**[Question 3] We will ask you about ASD diagnosis notification of pediatric patients (up to high school) over the past year (November 2014 to October 2015).**

A. How did you handle diagnosis notification of children themselves?

(after selecting one, answer Question A-2 or Question A-3)

**A-2.** This question is asked for those who responded with **answer 1 or answer 2**.

1. Notified of diagnosis as a general rule　　　　　　　Are there any exceptions? (select one)

2. Did not notify of diagnosis as a general rule　　　　1. No

3. Decided case-by-case　　　　　2. Yes

　 　 (Example: 　　 )

**A-3.** This question is asked for those who responded with **answer 3**.

Were the decision-making criteria for whether to notify or not notify of the diagnosis established 　in advance? (select one)

1. Not established

2. Established ⇒ Please write the specifics of the decision-making criteria (free response, format does not matter)

　　　　　　• When ( ) is ( ), diagnosis is **notified**.

　　　　　　　• When ( ) is ( ), diagnosis is **not notified**.

　　　　　　　• Other (　　　 　　　　　 　　　　　　　　　　　　　　　　　　　　　　)

B. Please select **all** terms you used when communicating the diagnosis to **children themselves**. **(can select multiple)**

1. Autism　　　　　　　2. Asperger’s (syndrome)　　　　　3. Pervasive developmental disorder

4. PDD　　　　5. Developmental disability　　　　　6. Autism spectrum

7. Autism spectrum disorder　　　　　　8. ASD

9. Other (　　　　　　　　　　　　　　　　　　　　)　　10. Did not communicate diagnosis

C. Please select **all** **reasons** that you communicated the diagnosis to **children themselves**. **(can select multiple)**

1. Because the diagnosis was confirmed　　　　　　　　　　　　　　2. Because treatment or services were started

3. Because they reached a certain age (age: years)　　　　　4. Because the parent(s) wanted it

5. Because the patient started to notice their differences from other people　　　　6. Because the patient asked

7. Other (　　　　　　　　　　　　　　　　　　　　)　　8. Did not communicate diagnosis

D. Were there times when you were unsure whether to communicate or not communicate the diagnosis to **the child themselves**? (select one)

1. Frequently　　　2. Sometimes　　　3. Rarely　　　4. Never

E. Those who have been unsure, please tell us specifically about what kinds of times you were unsure. (free response)

**[Question 4] We will ask you about the conditions/reasons for diagnosis notification of ASD to children (up to high school) themselves.**

A. How much do you emphasize the following items as conditions/reasons for **performing** **diagnosis notification** to **children themselves**? (for each item, select one number from 1 to 4)

|  | Conditions/reasons for performing **diagnosis notification** | 1  Emphasize | 2  If I had to say, emphasize | 3  If I had to say, do not emphasize | 4  Do not emphasize |
| --- | --- | --- | --- | --- | --- |
| 1 | The patient’s ability to understand is sufficient | 1 | 2 | 3 | 4 |
| 2 | The school understands about ASD | 1 | 2 | 3 | 4 |
| 3 | The patient’s ASD characteristics are strong | 1 | 2 | 3 | 4 |
| 4 | There are support services that the patient can receive | 1 | 2 | 3 | 4 |
| 5 | The patient’s treatment is necessary | 1 | 2 | 3 | 4 |
| 6 | The parent(s) want notification | 1 | 2 | 3 | 4 |
| 7 | Other medical care staff are cooperative | 1 | 2 | 3 | 4 |
| 8 | The patient asks their diagnosis | 1 | 2 | 3 | 4 |
| 9 | The relationship between the doctor and the patient is good | 1 | 2 | 3 | 4 |
| 10 | It is clear that the patient’s characteristics are within the diagnostic criteria | 1 | 2 | 3 | 4 |
| 11 | The symptoms of the patient’s secondary disorder are stable | 1 | 2 | 3 | 4 |
| 12 | The parent(s) understand about ASD | 1 | 2 | 3 | 4 |
| 13 | There is time to explain it to the patient | 1 | 2 | 3 | 4 |
| 14 | The patient is starting to notice that they are different from other people | 1 | 2 | 3 | 4 |
| 15 | The relationship between the parent(s) and the patient is good | 1 | 2 | 3 | 4 |
| 16 | The patient’s age is high | 1 | 2 | 3 | 4 |
| 17 | The patient has a right to know | 1 | 2 | 3 | 4 |
| 18 | The parents are in agreement | 1 | 2 | 3 | 4 |
| 19 | The patient’s ASD characteristics are weak | 1 | 2 | 3 | 4 |
| 20 | The parent(s) consent to notification | 1 | 2 | 3 | 4 |
| 21 | Please list any other conditions/reasons that you emphasize (　　　　　　　　　　　　　　　　　　　) | | | | |

B. How much do you emphasize the following items as conditions/reasons for **not performing** **diagnosis notification** to **children themselves**? (for each item, select one number from 1 to 4)

About two thirds of the way done

**Please proceed to the next page**

|  | Conditions/reasons for not performing **diagnosis notification** | 1  Emphasize | 2  If I had to say, emphasize | 3  If I had to say, do not emphasize | 4  Do not emphasize |
| --- | --- | --- | --- | --- | --- |
| 1 | The patient’s ability to understand is insufficient | 1 | 2 | 3 | 4 |
| 2 | The school does not understand about ASD | 1 | 2 | 3 | 4 |
| 3 | The patient’s ASD characteristics are weak | 1 | 2 | 3 | 4 |
| 4 | There are no support services that the patient can receive | 1 | 2 | 3 | 4 |
| 5 | The patient’s treatment is unnecessary | 1 | 2 | 3 | 4 |
| 6 | The parent(s) do not want notification | 1 | 2 | 3 | 4 |
| 7 | Other medical care staff are not cooperative | 1 | 2 | 3 | 4 |
| 8 | The patient does not ask their diagnosis | 1 | 2 | 3 | 4 |
| 9 | The relationship between the doctor and the patient is bad | 1 | 2 | 3 | 4 |
| 10 | It is not clear whether the patient’s characteristics are within the diagnostic criteria | 1 | 2 | 3 | 4 |
| 11 | The symptoms of the patient’s secondary disorder are unstable | 1 | 2 | 3 | 4 |
| 12 | The parent(s) do not understand about ASD | 1 | 2 | 3 | 4 |
| 13 | There is not time to explain it to the patient | 1 | 2 | 3 | 4 |
| 14 | The patient does not notice that they are different from other people | 1 | 2 | 3 | 4 |
| 15 | The relationship between the parent(s) and the patient is bad | 1 | 2 | 3 | 4 |
| 16 | The patient’s age is low | 1 | 2 | 3 | 4 |
| 17 | The patient has a right to not know | 1 | 2 | 3 | 4 |
| 18 | Disagreement between the parents is observed | 1 | 2 | 3 | 4 |
| 19 | The patient’s ASD characteristics are strong | 1 | 2 | 3 | 4 |
| 20 | The parent(s) are opposed to notification | 1 | 2 | 3 | 4 |
| 21 | Please list any other conditions/reasons that you emphasize (　　　　　　　　　　　　　　　　　　　) | | | | |

**[Question 5] We will ask you what you think about ASD.**

A. Was your diagnosis of ASD in everyday medical care impacted by the revision from DSM-IV to DSM-5? (select one)

1. Diagnosis became easier　　　　2. Did not change　　　　3. Diagnosis became harder

B. Do you think it’s best to change the diagnosis name ASD (autism spectrum disorder)? (select one)

1. I think it’s fine not to change it

2. Neither

3. I think it’s best to change it ⇒ (example:　　　　　　　　　　　　　　　　　　　　　　　)

C. How do you think it’s best, generally, to handle diagnosis notification of children themselves? (select one)

1. It’s best to provide diagnosis notification as a general rule

2. It’s best not to provide diagnosis notification as a general rule

3. It’s best to decide case-by-case

4. Other (　　　 　　　　　 　　　　　　　　　　　　　　　　　　　　　　)

D. When providing diagnosis notification to children themselves, generally, when do you think is best? (select one)

1. When the diagnosis is confirmed

2. When treatment (medication, hospitalization, etc.) or services (habilitation, special education classroom, etc.) are started

3. When the patient has reached a certain age (age: years)

4. When the parents want

5. When the patient has started to notice that they are different from other people

6. When the patient has asked

7. Other (　　　 　　　　　 　　　　　　　　　　　　　　　　　　　　　　)

8. It’s best to never provide diagnosis notification

E. When providing diagnosis notification to children themselves, generally, who do you think should inform the patient? (select one)

1. Parent (Guardian)　　　　　　　2. Doctor

3. A medical professional other than a doctor (　　　　　　　　　　　　　　　　　)

4. Other (　　　　　　　　　　　　　　　　　)

5. It’s best to never provide diagnosis notification

--- - --- - --- - --- - --- - --- - --- - --- - --- - --- - --- - --- - --- - --- - --- - --- - --- - --- - --- - --- - --- - --- - --- - --- ---

Only half a page left

**Please proceed to the next page**

F. There is a perspective that ASD is a personal characteristic, and a perspective that it is a disorder.

What do you think? (select one)

1. Completely a personal characteristic　　　　　　　　　　　2. If I had to say, a personal characteristic

3. If I had to say, a disorder　　　　　　4. Completely a disorder

G. Please answer the reason you think that response to Question F. (free response)

**[Question 6] Please write freely if you have any other opinions**

**concerning this questionnaire or concerning notification of ASD.**

------------------------------------------------------------------------------------------------------------------------------------

**That is all the questions.**

In the future, we are planning an interview survey to further deepen the results obtained in this survey. If you are willing to help, please fill out your contact information and select the ideal notification below.

　The contact information provided will only be used when asking for assistance with the interview, and privacy will be protected. Further, after we receive the questionnaire, the responses will be anonymized such that individuals cannot be identified, and the contact information provided will be removed.

　Even if contact information is provided, it does not automatically mean participation in future research. Later on, you can choose not to participate. Interviews themselves will be conducted at a time and place that is convenient for you.

Thank you very much for your cooperation.

--- - --- - --- - --- - --- - --- - --- - --- - --- - --- - --- - --- - --- - --- - --- - --- - --- - --- - --- - --- - --- - --- - --- - --- ---

| Pronunciation  Name |  |
| --- | --- |
| E-mail |  |
| Telephone number |  |
| Ideal notification (select one) | 1. Notify as a general rule 2. Do not notify as a general rule　3. Case-by-case |

**Thank you very much for your cooperation.**

After confirming that there are no missing answers,

please enclose in the self-addressed stamped envelope and return.
