# Supplementary material for: Disclosing a diagnosis of autism spectrum disorder without intellectual disability to pediatric patients in Japan in early diagnostic stages and associated factors: a cross-sectional study
Source: Biopsychosoc Med. 2022 Aug 20;16:18. doi: 10.1186/s13030-022-00247-0 (PMC9391641; doi:10.1186/s13030-022-00247-0)
Supplement: Supplementary file 2 — Additional file 2. [file 13030_2022_247_MOESM2_ESM.docx]

**Additional file 2**

Chi square goodness-of-fit test of distribution

|  | Society members  (N=1,995) | |  | Respondents  (N=612) | | Response rate | χ^2^ test  p-value |
| --- | --- | --- | --- | --- | --- | --- | --- |
|  | n | % |  | n | % | % |  |
| Sex |  |  |  |  |  |  | 0.437 |
| Male | 1,198 | 60.1 |  | 339 | 56.7 | 28.3 |  |
| Female | 797 | 39.3 |  | 259 | 43.3 | 32.5 |  |
| Not given | － | － |  | 14 | － | － |  |
| Specialty |  |  |  |  |  |  | 0.524 |
| Pediatrics | 322 | 16.1 |  | 120 | 20.2 | 37.3 |  |
| Psychiatry | 1,642 | 82.3 |  | 464^a^ | 78.0 | 28.3 |  |
| Other | 31 | 1.6 |  | 11 | 1.8 | 35.3 |  |
| Not given | － | － |  | 17 | － | － |  |
| Region |  |  |  |  |  |  | 0.997 |
| Hokkaido | 79 | 4.0 |  | 20 | 3.4 | 25.3 |  |
| Tohoku | 134 | 6.7 |  | 35 | 5.9 | 26.1 |  |
| Kanto | 611 | 30.6 |  | 184 | 30.9 | 30.1 |  |
| Chubu | 341 | 17.1 |  | 107 | 18.0 | 31.4 |  |
| Kinki | 423 | 21.2 |  | 135 | 22.7 | 32.0 |  |
| Chugoku | 127 | 6.4 |  | 37 | 6.2 | 29.1 |  |
| Shikoku | 74 | 3.7 |  | 14 | 2.3 | 19.0 |  |
| Kyushu | 206 | 10.3 |  | 64 | 10.7 | 31.0 |  |
| Not given | － | － |  | 16 | － | － |  |
| Age (years) |  |  |  |  |  |  | 0.998 |
| 26-30 | 45 | 2.3 |  | 8 | 1.4 | 17.8 |  |
| 31-35 | 181 | 9.2 |  | 51 | 8.6 | 28.2 |  |
| 36-40 | 283 | 14.4 |  | 97 | 16.4 | 34.3 |  |
| 41-45 | 268 | 13.7 |  | 89 | 15.0 | 33.2 |  |
| 46-50 | 258 | 13.2 |  | 91 | 15.4 | 35.3 |  |
| 51-55 | 295 | 15.1 |  | 93 | 15.7 | 31.5 |  |
| 56-60 | 257 | 13.1 |  | 63 | 10.6 | 24.5 |  |
| 61-65 | 170 | 8.7 |  | 46 | 7.8 | 27.1 |  |
| 66-70 | 103 | 5.3 |  | 27 | 4.6 | 26.2 |  |
| 71-75 | 57 | 2.9 |  | 13 | 2.2 | 22.8 |  |
| 76-80 | 24 | 1.2 |  | 7 | 1.2 | 29.2 |  |
| 81- | 18 | 0.9 |  | 7 | 1.2 | 38.9 |  |
| Not given | 36 | － |  | 20 | － | － |  |

a: Consists of 245 child psychiatrists, 214 adult psychiatrists, and 5 society members who responded “Other” but were determined to be psychiatrists.
